# Supplementing with L-Tryptophan Increases Medium Protein and Alters Expression of Genes and Proteins Involved in Milk Protein Synthesis and Energy Metabolism in Bovine Mammary Cells

**DOI:** 10.3390/ijms22052751

**Published:** 2021-03-09

**Authors:** Jay Ronel V. Conejos, Jalil Ghassemi Nejad, Jung-Eun Kim, Jun-Ok Moon, Jae-Sung Lee, Hong-Gu Lee

**Affiliations:** 1 Department of Animal Science and Technology, Konkuk University, Seoul 05029, Korea; jvconejos@up.edu.ph (J.R.V.C.); jalilgh@konkuk.ac.kr (J.G.N.); sumzzzing@gmail.com (J.-E.K.); jslee78@konkuk.ac.kr (J.-S.L.); 2 Institute of Animal Science, College of Agriculture and Food Sciences, University of the Philippines Los Baños, College Batong Malake, Los Baños, Laguna 4031, Philippines; 3 Institute of Integrated Technology, CJ CheilJedang, Suwon 16495, Korea; junok.moon@cj.net

**Keywords:** L-tryptophan, amino acids, MAC-T cell, proteomics, omics, β-casein, mTOR

## Abstract

The objective of this study was to investigate the effects of supplementing with L-tryptophan (L-Trp) on milk protein synthesis using an immortalized bovine mammary epithelial (MAC-T) cell line. Cells were treated with 0, 0.3, 0.6, 0.9, 1.2, and 1.5 mM of supplemental L-Trp, and the most efficient time for protein synthesis was determined by measuring cell, medium, and total protein at 0, 24, 48, 72, and 96 h. Time and dose tests showed that the 48 h incubation time and a 0.9 mM dose of L-Trp were the optimal values. The mechanism of milk protein synthesis was elucidated through proteomic analysis to identify the metabolic pathway involved. When L-Trp was supplemented, extracellular protein (medium protein) reached its peak at 48 h, whereas intracellular cell protein reached its peak at 96 h with all L-Trp doses. β-casein mRNA gene expression and genes related to milk protein synthesis, such as mammalian target of rapamycin (mTOR) and ribosomal protein 6 (RPS6) genes, were also stimulated (*p* < 0.05). Overall, there were 51 upregulated and 59 downregulated proteins, many of which are involved in protein synthesis. The results of protein pathway analysis showed that L-Trp stimulated glycolysis, the pentose phosphate pathway, and ATP synthesis, which are pathways involved in energy metabolism. Together, these results demonstrate that L-Trp supplementation, particularly at 0.9 mM, is an effective stimulus in β-casein synthesis by stimulating genes, proteins, and pathways related to protein and energy metabolism.

## 1. Introduction

Balancing the profile of essential amino acids (EAAs) can result in higher utilization efficiency of nitrogen, leading to enhanced bovine milk protein synthesis [1,2]. Due to the pivotal importance of milk protein in human health, investigating supplementation with AAs (e.g., methionine, lysine) as the main nutrients that can positively stimulate milk protein synthesis in mammary epithelial cells (MEC) has been a priority as of late [2]. Tryptophan (Trp), known as a conditional EAA [3], can be supplemented in the diets of animals and humans when targeting maximal production, cell growth, and proliferation [4,5,6]. Furthermore, Trp has been widely introduced as a mostly harmless supplement for humans to cope with health issues such as stress and depression. In animals of different species, L-Trp is used for alleviating stress (e.g., heat and cold) [4] by simulating serotonin [7] and melatonin [4,5,8], improving muscle cell growth [6], antioxidation [9], and inducing milk protein, lactose, and unsaturated fatty acids, which can also enhance human health [10,11], as people are the primary consumers of the products. While L-Trp as a supplemental nutrient has been used to induce muscle growth and development in human athletes [12,13] and beef cattle [6], there is a lack of insight into defining the optimum doses for enriching milk quality components to avoid the risk of overdose effects and provide maximum positive effects.

In monogastric cell lines and tissues, the stimulation of protein synthesis through mammalian target of rapamycin (mTOR) signaling is activated by individual AAs, in particular leucine (Leu) [2,14]. However, to what extent the mTOR signaling pathway can be activated directly by changes in EAAs, specifically L-Trp ratios, in bovine MEC remains unknown [2]. Moreover, the underlying mechanisms behind the effects of L-Trp need to be addressed. Before investigating the implication of L-Trp usage in vivo, it is important to define the optimum levels according to several prior in vitro studies in order to validate the benefits while avoiding ineffective high or low levels of supplementation.

In our laboratory, we took steps to examine the effects of L-Trp supplementation on muscle development and gene expression during heat and cold stress, and L-Trp proved to have anti-stress effects by simulating serotonin and melatonin in beef cattle [4,5,6]. Furthermore, in order to comprehend the mechanisms of L-Trp supplementation in influencing protein synthesis or affecting energy metabolism, metabolic pathways need to be investigated [10,12,13]. Investigating the metabolic pathway related to induced protein synthesis will help to improve our understanding of the reasons behind the phenomena [7,11,15], particularly for the purpose of milk enrichment. However, to date, no provisional and validated study has been conducted to determine what levels of L-Trp in a bovine mammary cell substratum will have the best influence on altered β-casein synthesis and related protein and energy metabolism pathways in vitro. L-Trp may supply nutrients necessary for protein synthesis, and this study may improve our understanding of how it regulates the expression of genes involved in milk protein synthesis in terms of nutrigenomics. Therefore, this study is the first to design supplementation of L-Trp in various doses to test the effects on increased medium protein and alterations in the expression of genes and proteins involved in milk protein synthesis and energy metabolism in bovine mammary cells.

## 2. Results

In this study, we compared the effects of different levels of L-Trp supplementation at various times on protein synthesis and the expression levels of β-casein, mRNA, and proteins.

### 2.1. AA Time and Dosage Sampling

**Figure 1:**
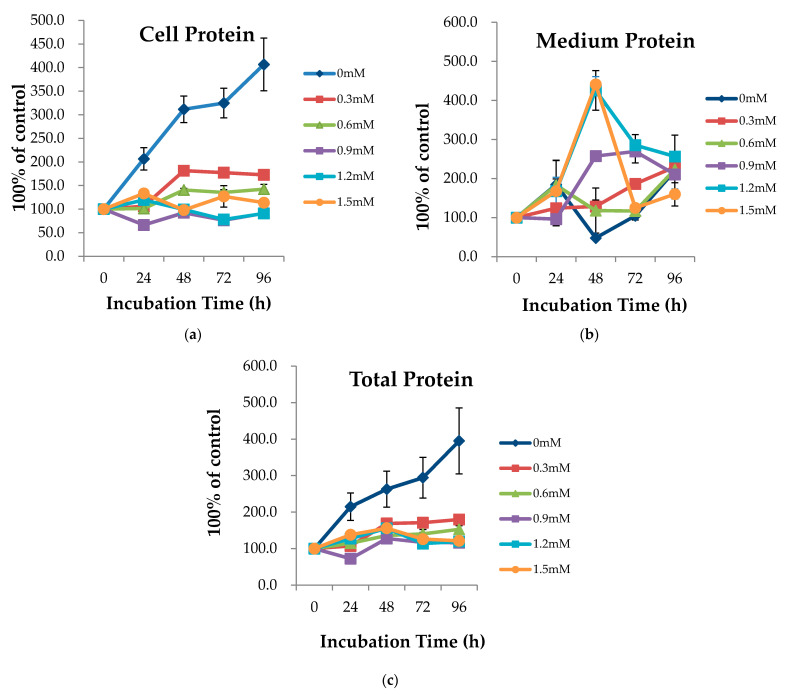
Relative protein content: (**a**) cell protein quantity, (**b**) medium protein quantity, and (**c**) total protein quantity (cell and medium) in bovine mammary epithelial (MAC-T) cells incubated with different levels of L-Trp (0, 0.3, 0.6, 0.9, 1.2, 1.5 mM) for 0, 24, 48, 72, and 96 h. Values are expressed as means ± SE (*n* = 6 per group).

Intracellular protein (cell protein) peaked at 96 h with 0 mM concentration of L-Trp (Figure 1a). In terms of extracellular protein (medium protein), when L-Trp was added, most concentrations peaked at 48 h (Figure 1b). Thus, 48 h was the optimal incubation time for the secretion of medium protein by bovine mammary epithelial (MAC-T) cells. The result of the incubation time test suggested that 48 h should be adopted as the incubation time for further tests of L-Trp efficacy for protein synthesis. Tables S1–S3 shows the average relative percentages of cell, medium and total protein, respectively. 

To have a clear picture of the distribution or spread of our data, the statistical analysis of heterogeneity (Q) was done on our cell, medium, and total protein (Tables S4–S6). Data show that the replications do not vary from each other or homogeneous (*p* < 0.05). 

**Figure 2:**
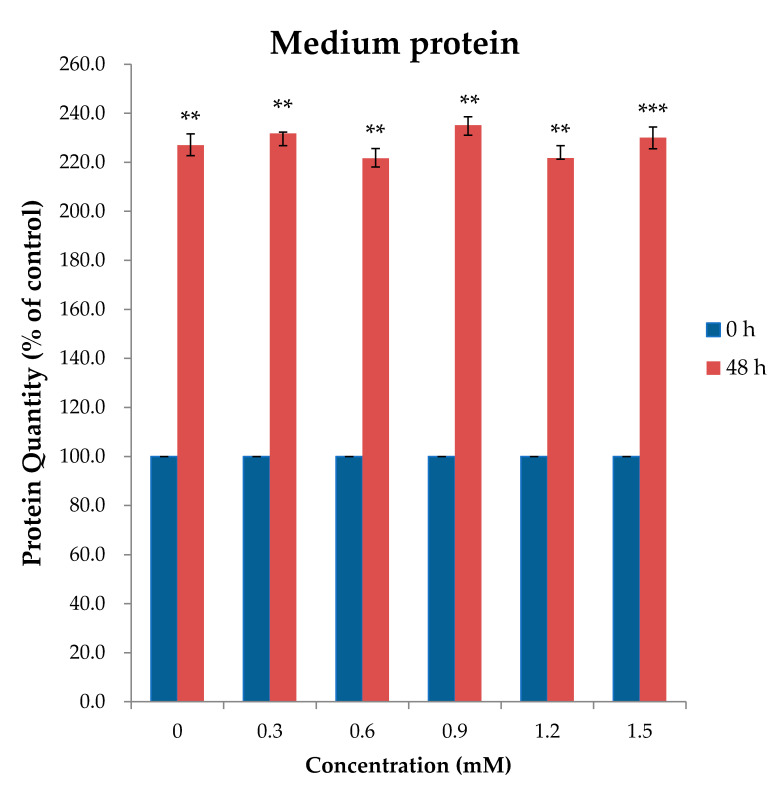
Relative medium protein content in MAC-T cells incubated with different levels of L-Trp (0, 0.3, 0.6, 0.9, 1.2, 1.5 mM) for 48 h. Values are expressed as means ± SE (*n* = 6 per group). ** < 0.01, *** < 0.001.

A confirmatory study was performed to determine the ideal dose of L-Trp at 48 h, which was considered as the optimal cultivation time (Figure 2). Although the value was not significantly different, 0.9 mM showed the numerically highest relative protein quantity. The relative percentage of medium protein level on MAC-T cells supplemented with different levels of L-Trp at 48 h incubation is shown on Table S7. Statistical analysis of heterogeneity (Q) of medium protein (Table S8) of MAC-T cells supplemented with different dosages (0, 0.3, 0.6, 0.9, 1.2, 1.5 mM) of L-Tryptophan at 48 h incubation time shows that replication samples are homogeneous except for 0.3 mM homogeneous (*p* < 0.05). 

### 2.2. Real-Time Polymerase Chain Reaction (RT-PCR)

**Figure 3:**
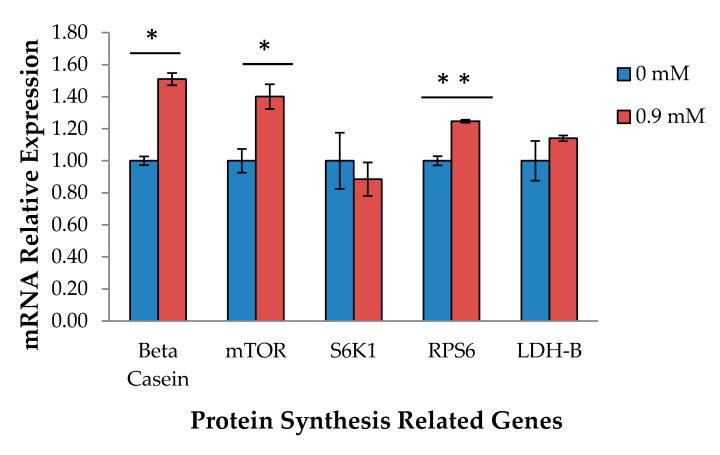
Changes in gene expression levels by addition of 0.9 mM L-Trp to MAC-T cells at 48 h. Analyzed by *t*-test between 0 and 0.9 mM L-Trp at 48 h: * *p* < 0.05, ** *p* < 0.01.

In terms of mRNA relative gene expression, β-casein mRNA expression and genes related to milk protein synthesis, such as *mTOR* and *RPS6*, were also stimulated upon addition of 0.9 mM L-Trp (*p* < 0.05) (Figure 3). On the other hand, there was no effect on *S6K1* and *LDH-B* gene expression upon addition of L-Trp (*p* > 0.05).

### 2.3. Proteome Analysis

**Figure 4:**
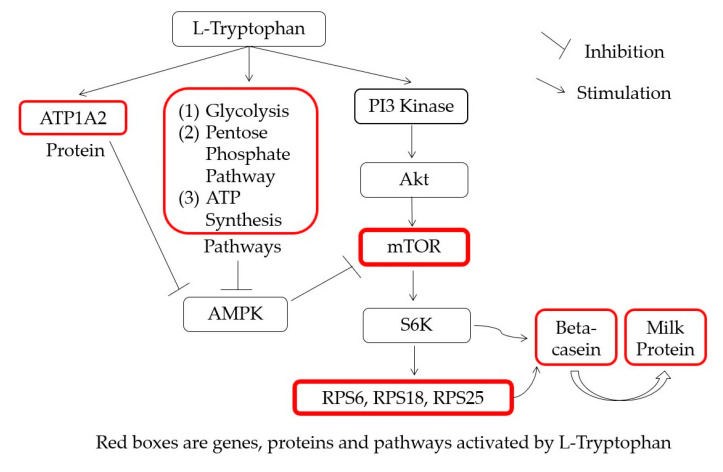
Diagram of the effect of L-Trp supplementation on milk protein synthesis pathways. ATP1A2, ATP1A2 sodium/potassium-transporting ATPase subunit alpha-2; AMPK, AMP-activated protein kinase; PI3 kinase, phosphoinositide 3-kinase; Akt, protein kinase B; mTOR, mammalian target of rapamycin; S6K, S6 kinase; RPS6, ribosomal protein S6; RPS18, 40S ribosomal protein S18; RPS25, 40S ribosomal protein S25.

In total, the addition of 0.9 mM L-Trp caused upregulation of 51 proteins and downregulation of 59 proteins, many of which are involved in protein synthesis (Table 1). The overall lists of upregulated as well as downregulated proteins in MAC-T cell are found in Tables S9 and S10. The result of protein pathway analysis showed that L-Trp stimulated glycolysis, the pentose phosphate pathway, and ATP synthesis, which are pathways involved in and related to energy metabolism (Table 2). Lastly, to summarize the results, a diagram of the effect of L-Trp supplementation on milk protein synthesis pathway was created (Figure 4).

## 3. Discussion

### 3.1. AA Time and Dosage Sampling

For the optimal time test, most concentrations peaked at 48 h, which was also the optimal incubation time for the secretion of medium protein by MAC-T cells. This is also the time when the secretion of β-casein was at its peak after L-Trp was added. The L-Trp dose test showed that 0.9 mM was the most effective concentration in increasing *CSN2* mRNA expression, indicating that this would be the optimal concentration level. This outcome suggests that 48 h should be selected to test different L-Trp concentrations because of the efficacy in increasing *CSN2* mRNA expression and protein synthesis in MAC-T cells in this time period.

### 3.2. CSN2 and Protein Synthesis-Related Gene Expression

Supplementation with 0.9 mM L-Trp also increased the relative expression of β-casein. In addition, L-Trp supplementation stimulated the mRNA relative expression of genes related to protein synthesis, especially *mTOR* and *RPS6*. A similar result was observed in a 9 h infusion study, in which a response in RPS6 phosphorylation to the addition of EAA plus glucose was reported [16]. Amino acids (AAs) not only act as substrates for protein synthesis, but also serve as signaling molecules that regulate synthesis [17,18]. The availability of AAs for mammary epithelial cells is of pivotal importance for the regulation of translation, and for the transport rate of AAs as one of the major limiting factors in protein synthesis [19,20,21]. In another study, a decline in mTOR signaling and fractional synthesis rate (FSR) in bovine mammary acini was observed when media were devoid of total EAA [22]. Hence, L-Trp may not only supply nutrients necessary for protein synthesis, but also regulate the expression of genes involved in milk protein synthesis in terms of nutrigenomics.

Specific AAs can affect translation initiation and elongation rates via two main pathways: the integrated stress response (ISR) and the mammalian target of rapamycin (mTOR) pathways [23]. There is direct evidence that AAs can increase mTOR phosphorylation and/or activity in the case of intact cells [24,25]. Amino acids affect mTOR signaling in bovine mammary epithelial (BME) cells [26], which is associated with increased milk protein synthesis in lactating cattle [16,27]. The stimulation of protein synthesis induced by AA [28] is known to be at least partially mediated by mTOR, a protein kinase present in rapamycin-sensitive mTOR complex 1 [29]. The mechanisms through which AA regulate mTOR stimulation are not yet fully understood, but it has been proposed that several protein factors, such as Ras homolog, class III PI3K Vps34, and Rag GTPases, mediate AA signaling on mTOR [30]. It is worth noting that among protein and energy metabolism-related pathways in this study, the Ras pathway was stimulated upon supplementation of L-Trp.

The mTOR pathway circles around mTOR complex 1 [31]. In mTOR complex 1, mTOR phosphorylates the downstream proteins that monitor the rate of translation initiation and elongation [31,32]. In previous studies, the effect of EAAs on the mTOR pathway was widely demonstrated in splanchnic, muscle, and mammary tissues [33,34,35]. When activated by AA, mTOR in turn catalyzes phosphorylation of S6K1 and 4EBP1 [36]. In the case of bovine mammary epithelial cells, a study showed that deprivation of all EAAs affected the phosphorylation of mTOR downstream proteins S6K1 and 4E-BP1 and fractional synthesis rates of β-LG [26]. This showed that infusion of EAAs plus glucose reduced the phosphorylation of the IRS-target eIF2 in mammary tissue and increased phosphorylation of the mTOR targets ribosomal S6 kinase 1 (S6K1) and S6 [16]. In addition, in another study, removal of L-Trp reduced S6K1 phosphorylation [37], which is consistent with a prior study in rat liver. Conversely, supplementing with L-Trp did not stimulate S6K1 gene expression, even though this is just expression and not phosphorylation. This is in accordance with the results of another study [38], in which Trp, Phe, and Met addition had no effect on S6K1 phosphorylation.

### 3.3. Proteomics Analysis

In the proteomics result, supplementation with L-Trp decreased EEF1A1, EEF2, and EEF1G protein expression. Although eEF2 protein is not a direct substrate of mTOR, an inverse relationship between phosphorylation of mTOR and eEF2 has been reported [39]. When eEF2 binds to the ribosome, eEF2 mediates the translocation step of the elongation process. Phosphorylation of eEF2 at Thr56 by eEF2 kinase decreases the affinity of eEF2 for the ribosome [40]. eEF2 phosphorylation, which decreases the elongation rate, is inhibited by the mTOR pathway downstream of kinase S6K1, which results in a positive effect of mTOR on the casein fractional synthesis rate (CFSR) [23], in line with the results obtained in this study. It has been suggested that eEF2 may be a limiting factor in milk protein synthesis [40]. The negative relationship between phosphorylated eEF2 and protein synthesis rates indicates an important role for eEF2 in mammary protein synthesis [37]. Another study also showed an inverse relationship between eEF2 phosphorylation and milk protein yield in dairy cows treated with growth hormone [12]. It has been reported that protein synthesis rates were strongly associated with phosphorylation of eEF2 in mammary tissue slices [37]. The aforementioned pathway review may explain the obtained results.

RPS6 was also negatively correlated with eEF2 [8]. This also agrees with our result that there was increased gene expression of RPS6 but decreased protein expression of eEF2. It has long been known that the addition of a physiological mixture of AAs to hepatocytes will result in strong and rapid phosphorylation of RPS6 [41,42]. RPS6 protein is a component of the 40S ribosomal subunit and one of the endpoints of insulin signaling, and phosphorylation of this protein is needed for the translation of certain mRNA molecules encoding proteins in the protein-translation machinery [30]. The increased expression of RPS6 coincides with the increased mTOR gene expression. This is in accordance with a previous study in which the AA-stimulated phosphorylation of RPS6 was completely inhibited by applying rapamycin, implying that mTOR3 is upstream of RPS6 in the AA signaling pathway [41,42].

It is also especially important to note the decreased expression of eukaryotic initiation factor 4A-I (EIF4A1) protein. Protein synthesis rates primarily depend on translation initiation and elongation rates, which are regulated by several eukaryotic initiation factors (eIFs) and elongation factors (eEFs) [43]. Activation of these proteins occurs through changes in their phosphorylation state, influenced by the addition of AAs and hormones [44]. Accordingly, studies have shown that AAs are likely to regulate eIF and eEF phosphorylation in skeletal muscle and liver cells via the mTOR signaling pathway [33,45].

### 3.4. Metabolic Pathway Analysis

Protein and energy metabolism-related pathway results showed that glycolysis, the pentose phosphate pathway, and ATP synthesis were stimulated upon supplementation with L-Trp. These energy-related pathways are stimulated in parallel with the increased protein synthesis rates due to the fact that milk protein synthesis is an energy-consuming process [46,47]. The interaction between energy and protein supplementation in the phosphorylation of mTOR in dairy cows infused for 36 h with starch and energy has been reported [48]. In that study, they investigated signaling pathways responsive to casein and starch infusion in primiparous mid-lactation Holstein cows. The results showed that cell signaling molecules involved in the regulation of milk protein synthesis responded differently to the various nutritional stimuli, and the phosphorylation of mTOR was increased in response to starch when casein was infused.

## 4. Materials and Methods

### 4.1. AA Dose and Sampling Time

Immortalized mammary epithelial (MAC-T) cells [38] from McGill University, Canada, were grown in 10 cm dishes (TPP, Trasadingen, Switzerland). MAC-T cells were incubated in DMEM/F12 basic medium for 72 h or until the cells reached 90% confluency. The measurement of doubling time of MAC-T cell and the method of determining cells reaching 90% confluency are found in Figures S1 and S2. The cells were then seeded into 6-well U-shaped multiwall plates (BD Falcon™, Franklin Lakes, NJ, USA). They were cultured in DMEM/F12 basic medium (Thermo Scientific, South Logan, UT, USA) supplemented with 10% fetal bovine serum (FBS), 100 units/mL penicillin/streptomycin (Thermo Scientific, South Logan, UT, USA), 5 μg/mL insulin, 1 μg/mL hydrocortisone, and 50 μg/mL gentamycin (Sigma-Aldrich Corp., St. Louis, MO, USA) at 37 °C in a 5% CO_2_ incubator [49,50]. When MAC-T cells reached 90% confluence, DMEM/F12 basic medium was replaced with DMEM/F12 lactogenic medium (without FBS) to differentiate MAC-T cells into β-casein (CSN2) secreting cells for 72 h. This medium contained 5 μg/mL bovine insulin, 1 μg/mL hydrocortisone, 100 units/mL penicillin/streptomycin, 50 μg/mL gentamycin, and 5 μg/mL prolactin (Sigma-Aldrich Corp., St. Louis, MO, USA) (Wang et al., 2014, 2015). The complete amino acid profile of lactogenic medium is listed in Table S11. After cell differentiation, a preliminary experiment was performed for the time and dose testing. Cells were treated with 0, 0.3, 0.6, 0.9, 1.2, and 1.5 mM of supplemental L-Trp, and the most efficient time for protein synthesis was determined by measuring cell, medium, and total protein at 0, 24, 48, 72, and 96 h. Then, a confirmatory experiment was performed to determine the ideal dose of L-Trp in the determined optimal cultivation time. Different doses of L-Trp (0, 0.3, 0.6, 0.9, 1.2, and 1.5 mM) were incubated at 48 h, which is the optimal time for protein synthesis. In all experiments, each treatment was replicated 6 times.

### 4.2. RNA Extraction and cDNA Synthesis

Total RNA was extracted from MAC-T cells using TRIzol^®^ (Life Technologies Corp., Carlsbad, CA, USA). The RNA quality and quantity were measured using a NanoDrop 1000^®^ Spectrophotometer with RNA-40 module (Thermo Fisher Scientific, Wilmington, DE, USA). RNA integrity number (RIN) was determined using an Agilent 2100 Bioanalyzer (Agilent Technologies Inc., Santa Clara, CA, USA). Then cDNA was prepared using an iScript cDNA synthesis kit (Bio-Rad Laboratories, Inc., Foster City, CA, USA) according to the manufacturer’s instructions. After incubating at 25 °C for 5 min, 42 °C for 30 min, and 85 °C for 5 min, the cDNA was quantified using the ssDNA-33 module of the Thermo NanoDrop 1000^®^ Spectrophotometer (Thermo Fisher Scientific, Wilmington, DE, USA).

### 4.3. Real-Time Polymerase Chain Reaction (RT-PCR)

Real-time PCR (RT-PCR) analysis was performed using a T100™ Thermal Cycler System. ACTB was used as the reference gene. Validated RT-PCR oligonucleotide primer sequences of forward and reverse primers specific for target genes were as follows: *CSN2* forward, 5′-AAATCTGCACCTTCCTCTGC-3′; *CSN2* reverse, 5′-GAACAGGCAGGACTTTGGAC-3′; *ACTB* forward, 5′-GCATGGAATCCTGCGGC-3′; *ACTB* reverse, 5′-GTAGAGGTCCTTGCGGATGT-3′. RT-PCR reactions were performed by initial incubation at 95 °C for 3 min followed by 50 cycles of denaturation at 95 °C for 10 s, annealing at specific temperature for 15 s (bovine CSN2 at 55 °C), and extension at 72 °C for 30 s (Table S12). RT-PCR analysis was conducted using the threshold cycle (2-ΔΔCT method) [51] to analyze relative gene expression changes from real-time quantitative PCR experiments. Relative quantification of expression levels of target genes in the treatment group was compared to the untreated group.

### 4.4. Protein Extraction and Quantification

After incubation in the treatment medium (lactogenic medium plus L-Trp) for 72 h, the culture medium was collected from adherent cells to determine protein quantity. All treatments were done with six replicates. The culture medium was centrifuged at 300× *g* for 5 min at 4 °C. The supernatant was transferred to a new tube for protein quantification using a Pierce BCA Protein Assay Kit (Pierce Biotechnology, Inc., Rockford, IL, USA) according to the manufacturer’s instructions. Cells were washed twice with ice cold 1 × PBS and then 200 μL cell lysis buffer containing 10 mM Tris/HCl, pH 8.3, 8 M urea, 5 mM EDTA, 4% CHAPS, and 1× protease inhibitor cocktail (GE Healthcare, Piscataway, NJ, USA) were added. The cell lysates were incubated at room temperature for 30 min and centrifuged at 21,952× *g* for 30 min at 20 °C. The supernatant was collected and stored at –80 °C until analysis.

### 4.5. Proteome Analysis

Cellular proteins were extracted using cell lysis buffer containing 20 mM Tris, 10 mM KCl, 1.5 mM MgCl2, 0.5 mM EDTA, 0.1% sodium dodecyl sulfate (SDS), and complete EDTA-free Protease Inhibitor Cocktail (Roche, Basel, Switzerland) after washing twice with ice-cold 1× PBS. Cell lysates were incubated at 4 °C for 30 min and centrifuged at 13,000× *g* for 10 min at 4 °C [41]. Then, supernatant was collected and stored at –80 °C until analysis. All treatments were replicated three times.

For proteome analysis, 100 μg of cell lysate protein was resuspended in 0.1% SDS in 50 mM triethyl ammonium bicarbonate (TEABC), pH 8.0. Proteins were chemically denatured using 10 mM tris (2-carboxyethyl) phosphine (TCEP) at 60 °C for 30 min and alkylated with 50 mM methyl methanethiosulfonate (MMTS) at room temperature for 30 min in the dark. Proteolytic digestion was conducted using trypsin (protein:trypsin = 50:1, g/g) overnight at 37 °C. Digested peptides were desalted and concentrated, then subjected to liquid chromatography (LC) tandem mass spectrometry (MS/MS) analysis. Total peptides were analyzed by nano-ultra-performance LC–MS/electrospray ionization quadrupole time-of-flight (UPLC–MS/ESI–Q–TOF) (Waters, Manchester, UK). LC peptide separation was performed using the nano Acquity system equipped with a Symmetry C18 5 μm, 5 mm × 300 μm pre-column and CSH C18 1.7 μm, 25 cm × 75 μm analytical column (Waters). Samples were separated using a 3–40% gradient mob Leu phase B (0.1% formic acid in acetonitrile) at a flow rate of 300 nL/min, followed by a 20 min rinse with 90% mob Leu phase B.

Data-dependent analysis (DDA) was performed to obtain two analytical replicates for each of three biological sets. This method is used to read a full MS scan in an m/z range of 400–1600 every 0.5 s and MS/MS scans (m/z range: 100–1990) every 0.5 s for the three most intense ions among the full-scan MS. Protein identification was performed by comparison with the International Protein Index (IPI) bovine database (v. 3.73; 30,403 entries) using the MASCOT search engine v. 2.4 (Matrix Science, Boston, MA, USA), using trypsin as the digestion enzyme, with a parent ion tolerance of 0.2 Da and fragment ion mass tolerance of 0.1 Da. Two missed cleavages were allowed during trypsin digestion. Oxidation (Met) and Methylthio (Cys) were specified as the modification settings. Proteins identified with >95% probability were filtered out. To evaluate the false discovery rate (FDR) of protein identification, data were searched against a combined database of normal and decoy data created by MASCOT. The FDR of all experiments in this study was <1%. The emPAI score of each protein was used to calculate its relative ratio [52]. The emPAI-based abundances in comparison with the actual values were within 63% on average, and this is similar or better than determination of abundance by protein staining [52].

### 4.6. Statistical Analysis

Statistical analysis (protein quantification data, *n* = 6; proteomics data, *n* = 3; quantitative real-time PCR data, *n* = 6) was conducted for significance testing using SAS v. 9.4 software (SAS Institute, Cary, NC, USA). Data were analyzed using Student’s *t*-test. Mean difference was considered statistically significant at *p* < 0.05. The experimental model was

Y_ijk_ = μ + τ_i_ + ε_ijk_,

where μ is the grand mean, τi is the L-Trp effect, and ε_ijk_ is the error variability.

Upregulated or downregulated proteins were detected for significance using the semi-quantification relative ratio (≥2 or ≤0.5), and detected proteins were analyzed using the PANTHER database (http://www.pantherdb.org accessed on 19 August 2020) for pathway analysis (*Bos taurus*).

## 5. Conclusions

Altogether, 0.9 mM L-Trp supplementation was found to be the optimum dose for stimulating medium protein and β-casein mRNA expression by stimulating the expression of genes related to milk protein synthesis and the increased production of proteins involved in energy metabolism and protein synthesis pathways. Protein and energy metabolism related pathways were also upregulated in the L-Trp treatment, eventually causing increased protein concentration in the MAC-T cell medium supplemented with L-Trp. In conclusion, L-Trp, particularly at 0.9 mM, was effective in increasing protein synthesis in MAC-T cells in vitro by stimulating the genes, proteins, and protein synthesis-related pathways involved in energy and protein synthesis. Also, our findings suggest that L-Trp may not only supply nutrients necessary for protein synthesis, but also regulate the expression of genes involved in milk protein synthesis in terms of nutrigenomics.

## Figures and Tables

**Table 1:** Differentially expressed proteins in MAC-T cells supplemented with L-Trp compared with control.

Detected Proteins		
Number of protein increased		51
Number of protein decreased		59
Selected downregulated and upregulated proteins		▲ ▼
HSPD1 (60 kDa heat shock protein, mitochondrial)	HSPD1	▲
HSPA1A (Heat shock 70 kDa protein 1A)	HSPA1A	▲
ATP5B (ATP synthase subunit beta, mitochondrial)	ATP5B	▼
EEF1A1 (Elongation factor 1 alpha 1)	EEF1A1	▼
RPSA (Similar to 40S ribosomal protein SA (fragment))	RPSA	▼
ATP synthase subunit alpha, mitochondrial	ATP5A1	▼
RPS18 (40S ribosomal protein S18)	RPS18	▲
EIF4A1 (Eukaryotic initiation factor 4A-I)	EIF4A1	▼
EEF2 (Elongation factor 2)	EEF2	▼
RPS25 (40S ribosomal protein S25)	RPS25	▲
EEF1G (Elongation factor 1-gamma)	EEF1G	▼
RPN2 (Dolichyl-diphosphooligosaccharide–protein glycosyltransferase)	RPN2	▼
ATP1A2 (Sodium/potassium-transporting ATPase subunit alpha-2)	ATP1A2	▼
GPI (Glucose-6-phosphate isomerase)	GPI	▼
RPL11 (60S ribosomal protein L11)	RPL11	▼
RPS2 (40S ribosomal protein S2)	RPS2	▼

▲, Upregulated (>2-fold greater protein expression than control); ▼, downregulated (<0.5-fold greater protein expression than control).

**Table 2:** Protein and energy metabolism-related pathways stimulated by supplementation of L-Trp compared with control.

Detected Pathways *
Apoptosis signaling pathway
p53 pathway
Glycolysis
Pentose phosphate pathway
ATP synthesis
CCKR signaling map
Endothelin signaling pathway
FGF signaling pathway
Ras pathway
EGF receptor signaling pathway

* Significantly increased protein and energy metabolism-related pathways (*p* < 0.05) compared with control, as determined by the PANTHER online tool for *Bos taurus* (see Methods for data explanation).

## Data Availability

Data will be available upon request.

## Supplementary Materials

The following are available online at https://www.mdpi.com/1422-0067/22/5/2751/s1.

## Abbreviations

CSN2	Beta-casein (β-casein)
ATP1A2	ATP1A2 Sodium/potassium-transporting ATPase subunit alpha-2
AMPK	AMP-activated protein kinase
PI3 Kinase	Phosphoinositide 3-kinase
Akt	Protein kinase B
mTOR	mammalian target of rapamycin
S6K	S6 Kinase
RPS6	Ribosomal protein S6
RPS18	40S ribosomal protein S18
RPS25	40S ribosomal protein S25
