# Supplementary material for: Supplementing with L-Tryptophan Increases Medium Protein and Alters Expression of Genes and Proteins Involved in Milk Protein Synthesis and Energy Metabolism in Bovine Mammary Cells
Source: Int J Mol Sci. 2021 Mar 9;22(5):2751. doi: 10.3390/ijms22052751 (PMC7963161; doi:10.3390/ijms22052751)
Supplement: Supplementary file 1 [file ijms-22-02751-s001.pdf]

Supplementary Material

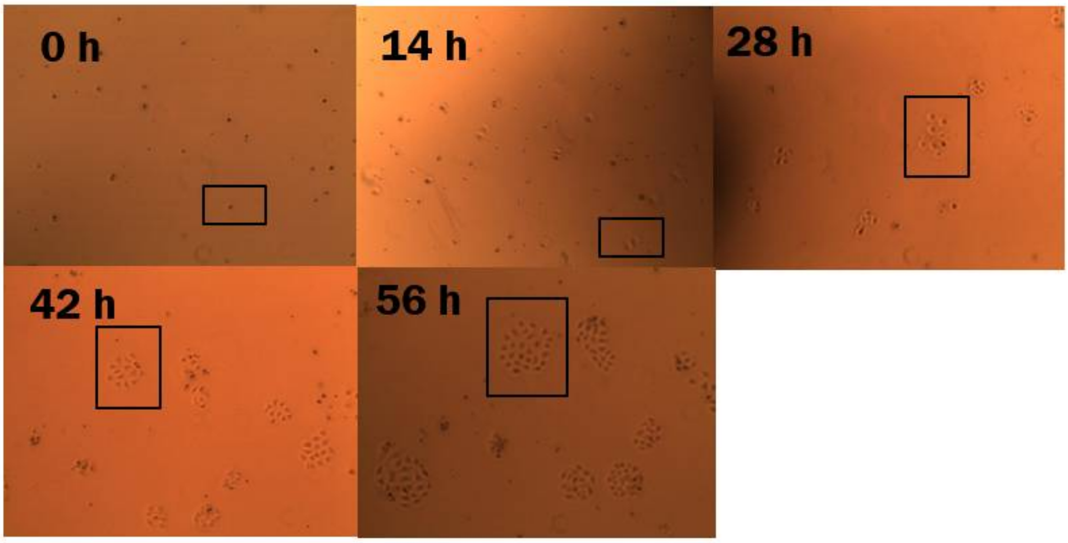

Time after seeding	0 h	14 h	28 h	42 h	56 h	Division Times	Doubling Time
Cell number	1	2	8	16	28	3.75	14.9 h

Figure S1. Doubling time of MAC-T cell in 10-cm round plate supplemented with the basic growth medium at 0, 14, 28, 42 and 56 h.

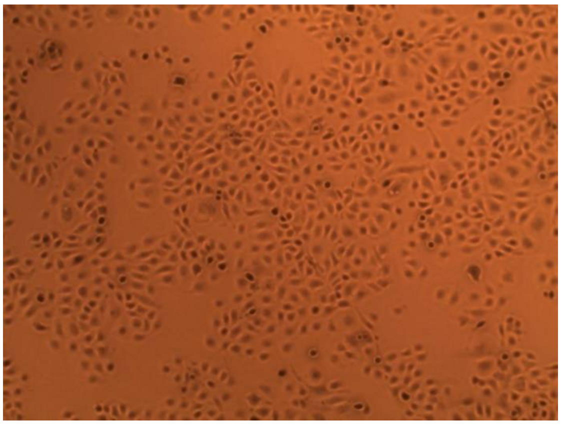

Figure S2. MAC-T cell with about 50% confluency. When the cell confluency reached about 50% confluency, considering the doubling time characteristics of our cell, the amount on time (h) wherein our cells will reach our target confluency can be estimated. For example, if our target confluency is around 90%, with a doubling time of 14.9 h, our cell will reach each confluency after 13.41 h after the cell reached confluency of 50%.

Table S1. Average relative percentage of cell protein level on MAC-T cells stimulated by L-Tryptophan supplementation.

	Incubation Time, h				
Concentration, mM	0	24	48	72	96
0	100.0	206.5	311.6	324.8	406.7
0.3	100.0	106.2	181.3	177.3	172.6
0.6	100.0	101.3	140.6	135.4	142.4
0.9	100.0	66.3	92.2	76.4	91.0
1.2	100.0	119.4	99.2	78.0	90.8
1.5	100.0	133.3	98.0	126.9	113.8

Table S2. Average relative percentage of medium protein level on MAC-T cells stimulated by L-Tryptophan supplementation.

	Incubation Time, h				
Concentration, mM	0	24	48	72	96
0	100.0	184.7	47.9	104.8	220.3
0.3	100.0	124.3	128.3	186.5	229.8
0.6	100.0	183.2	118.0	116.9	223.0
0.9	100.0	96.2	257.7	269.5	210.7
1.2	100.0	167.9	425.7	285.5	256.6
1.5	100.0	168.1	441.3	125.0	160.3

Table S3. Average relative percentage of total (cell and medium) protein level on MAC-T cells stimulated by L-Tryptophan supplementation.

	Incubation Time, h				
Concentration, mM	0	24	48	72	96
0	100.0	214.8	262.9	294.4	395.0
0.3	100.0	107.2	168.8	171.0	179.7
0.6	100.0	114.8	135.9	140.2	152.8
0.9	100.0	72.5	127.6	117.0	116.0
1.2	100.0	127.3	155.8	113.7	119.3
1.5	100.0	138.1	155.9	125.9	121.3

Table S4. Statistical analysis of heterogeneity (Q) of cell protein of MAC-T cells supplemented with different dosages (0, 0.3, 0.6, 0.9, 1.2, 1.5 mM) of L-Tryptophan at 0, 24, 48, 72 and 96 h incubation time.

**Cell Protein**	**Possible Outlier**	**Gap**	**Range**	**Q**	**Confidence Interval**	**Critical Value**	**Outlier Y/N**
0mM							
24	145.3	88.2	95.5	**0.92**	95%	0.97	N
48	244.7	76.7	124.2	**0.62**	95%	0.97	N
72	399.9	86.0	139.5	**0.62**	95%	0.97	N
96	267.2	178.6	239.8	**0.74**	95%	0.97	N
0.3mM							
24	118.2	17.6	18.4	**0.96**	95%	0.97	N
48	187.5	7.6	11.0	**0.70**	95%	0.97	N
72	190.7	17.9	22.5	**0.80**	95%	0.97	N
96	174.8	2.6	4.1	**0.64**	95%	0.97	N
0.6mM							
24	111.7	11.3	19.8	**0.57**	95%	0.97	N
48	147.6	16.1	11.4	**1.42**	95%	0.97	Y
72	146.8	16.1	11.4	**1.42**	95%	0.97	Y
96	122.2	30.0	18.0	**1.66**	95%	0.97	Y
0.9mM							
24	53.8	18.5	19.1	**0.96**	95%	0.97	N
48	98.5	9.2	9.6	**0.96**	95%	0.97	N
72	70.7	8.3	8.8	**0.95**	95%	0.97	N
96	83.3	10.3	13.0	**0.79**	95%	0.97	N
1.2mM							
24	108.9	12.8	18.8	**0.68**	95%	0.97	N
48	107.9	12.3	13.7	**0.90**	95%	0.97	N
72	71.8	8.4	10.3	**0.82**	95%	0.97	N
96	94.8	5.1	6.9	**0.73**	95%	0.97	N
1.5mM							
24	148.1	18.0	26.4	**0.68**	95%	0.97	N
48	110.3	16.1	20.8	**0.78**	95%	0.97	N
72	85.8	45.1	78.2	**0.58**	95%	0.97	N
96	118.8	5.3	9.8	**0.54**	95%	0.97	N

Table S5. Statistical analysis of heterogeneity (Q) of medium protein of MAC-T cells supplemented with different dosages (0.3, 0.6, 0.9, 1.2, 1.5 mM) of L-Tryptophan at 0, 24, 48, 72 and 96 h incubation time.

**Medium Protein**	**Possible Outlier**	**Gap**	**Range**	**Q**	**Confidence Interval**	**Critical Value**	**Outlier Y/N**
0.3mM							
24	34.3	129.1	140.9	**0.92**	95%	0.97	N
48	149.0	20.7	41.3	**0.50**	95%	0.97	N
72	194.1	7.6	15.2	**0.50**	95%	0.97	N
96	260.3	15.2	59.4	**0.26**	95%	0.97	N
0.6mM							
24	260.2	77.0	154.0	**0.50**	95%	0.97	N
48	47.5	70.5	141.1	**0.50**	95%	0.97	N
72	121.2	4.3	99.1	**0.04**	95%	0.97	N
96	224.0	1.0	7.6	**0.13**	95%	0.97	N
0.9mM							
24	93.4	3.1	5.6	**0.55**	95%	0.97	N
48	236.0	30.2	70.5	**0.43**	95%	0.97	N
72	251.4	21.5	97.3	**0.22**	95%	0.97	N
96	253.2	59.1	68.3	**0.87**	95%	0.97	N
1.2mM							
24	109.9	66.5	107.3	**0.62**	95%	0.97	N
48	526.2	140.3	161.2	**0.87**	95%	0.97	N
72	234.2	61.8	92.1	**0.67**	95%	0.97	N
96	281.8	36.9	38.7	**0.95**	95%	0.97	N
1.5mM							
24	237.3	91.1	116.7	**0.78**	95%	0.97	N
48	478.2	44.7	161.2	**0.28**	95%	0.97	N
72	138.1	16.2	92.1	**0.18**	95%	0.97	N
96	186.2	35.9	41.8	**0.86**	95%	0.97	N

Table S6. Statistical analysis of heterogeneity (Q) of total protein of MAC-T cells supplemented with different dosages (0, 0.3, 0.6, 0.9, 1.2, 1.5 mM) of L-Tryptophan at 0, 24, 48, 72 and 96 h incubation time.

**Total Protein**	**Possible Outlier**	**Gap**	**Range**	**Q**	**Confidence Interval**	**Critical Value**	**Outlier Y/N**
0mM							
24	288.1	96.2	123.9	**0.78**	95%	0.97	N
48	361.6	147.0	149.2	**0.99**	95%	0.97	Y
72	405.9	165.8	168.7	**0.98**	95%	0.97	Y
96	573.3	239.2	295.5	**0.81**	95%	0.97	N
0.3mM							
24	108.7	0.9	3.6	**0.26**	95%	0.97	N
48	183.3	17.5	25.9	**0.68**	95%	0.97	N
72	189.4	17.2	38.2	**0.45**	95%	0.97	N
96	176.7	4.0	5.2	**0.76**	95%	0.97	N
0.6mM							
24	93.4	23.7	40.5	**0.59**	95%	0.97	N
48	128.0	10.0	13.7	**0.73**	95%	0.97	N
72	164.1	35.4	36.1	**0.98**	95%	0.97	Y
96	133.2	26.8	31.9	**0.84**	95%	0.97	N
0.9mM							
24	62.0	14.9	16.6	**0.90**	95%	0.97	N
48	136.9	12.2	15.7	**0.78**	95%	0.97	N
72	112.6	4.8	8.4	**0.58**	95%	0.97	N
96	119.9	5.6	6.0	**0.94**	95%	0.97	N
1.2mM							
24	136.5	10.7	16.9	**0.63**	95%	0.97	N
48	171.5	20.0	26.9	**0.74**	95%	0.97	N
72	108.2	7.4	9.1	**0.81**	95%	0.97	N
96	124.1	6.6	8.0	**0.83**	95%	0.97	N
1.5mM							
24	126.0	16.9	19.6	**0.86**	95%	0.97	N
48	167.8	13.5	22.0	**0.61**	95%	0.97	N
72	93.4	34.5	63.2	**0.55**	95%	0.97	N
96	124.4	3.1	5.0	**0.61**	95%	0.97	N

Table S7. Relative percentage of medium protein level on MAC-T cells supplemented with different levels of L-Tryptophan at 48 h incubation.

Concentration, mM	Incubation Time, h	
	0	48
0	0.0	226.9
0	100.0	221.4
0	100.0	232.6
**Avg**	**100.0**	**227.0**
**STD**	**57.7**	**5.6**
**SE**	**33.3**	**3.2**
0.3	100.0	230.8
0.3	100.0	232.3
0.3	100.0	232.3
**Avg**	**100.0**	**231.8**
**STD**	**0.0**	**0.9**
**SE**	**0.0**	**0.5**
0.6	100.0	229.5
0.6	100.0	216.2
0.6	100.0	219.1
**Avg**	**100.0**	**221.6**
**STD**	**0.0**	**7.0**
**SE**	**0.0**	**4.0**
0.9	100.0	236.2
0.9	100.0	228.6
0.9	100.0	240.5
**Avg**	**100.0**	**235.1**
**STD**	**0.0**	**6.0**
**SE**	**0.0**	**3.5**
1.2	100.0	217.8
1.2	100.0	215.7
1.2	100.0	231.7
**Avg**	**100.0**	**221.7**
**STD**	**0.0**	**8.7**
**SE**	**0.0**	**5.0**
1.5	100.0	224.8
1.5	100.0	235.4
1.5	100.0	
**Avg**	**100.0**	**230.1**
**STD**	**0.0**	**7.5**
**SE**	**0.0**	**4.3**

Table S8. Statistical analysis of heterogeneity (Q) of medium protein of MAC-T cells supplemented with different dosages (0, 0.3, 0.6, 0.9, 1.2, 1.5 mM) of L-Tryptophan at 48 h incubation time.

Concentration (mM	Possible Outlier	Gap	Range	Q	Confidence Interval	Critical Value	Outlier Y/N
0	221.4	5.5	11.2	**0.49**	95%	0.97	N
0.3	230.8	1.5	1.5	**1.00**	95%	0.97	Y
0.6	229.5	10.4	13.3	**0.78**	95%	0.97	N
0.9	228.6	7.6	223.8	**0.03**	95%	0.97	N
1.2	231.7	14.0	16.0	**0.87**	95%	0.97	N
1.5	235.4	5.3	10.6	**0.50**	95%	0.97	N

Table S9. List of upregulated proteins in MAC-T Cell supplemented with L-Tryptophan

	**Protein ID**	**Protein Name**	**Score**		**%emPAI (Semi quantification)**	
			**Con**	**Trp**	**Con**	**Trp**
1	IPI00712562	ACTN1 Alpha-actinin-1		514		0.28
2	IPI00826297	HSPD1 60 kDa heat shock protein, mitochondrial		280		0.81
3	IPI00700035	HSPA1A Heat shock 70 kDa protein 1A		377		0.17
4	IPI00702028	TAGLN2 Transgelin-2		476		1.57
5	IPI00715354	SFN 14-3-3 protein sigma	85	515	0.67	1.78
6	IPI00701086	HIST1H2BD Histone H2B		370		2.47
7	IPI00838361	HSPB1 Uncharacterized protein	66	284	0.37	0.6
8	IPI00694304	CLU Clusterin	38	268	0.07	0.15
9	IPI00706942	TPI1 Triosephosphate isomerase	31	327	0.14	0.95
10	IPI00686173	GSTP1 Glutathione S-transferase P	89	158	0.16	0.35
11	IPI01002854	LOC100337473 GJ17461-like	118	139	0.56	0.95
12	IPI00709865	RPSA 40S ribosomal protein SA		90		0.24
13	IPI00732001	SPTAN1 Uncharacterized protein		87		0.04
14	IPI00827006	HIST1H1D Uncharacterized protein		94		0.17
15	IPI00689850	RPS18 40S ribosomal protein S18		123		0.48
16	IPI00867349	CAST protein		73		0.09
17	IPI00686735	LASP1 LIM and SH3 domain protein 1		53		0.13
18	IPI00699333	ANXA8L1 Annexin A8	51	77	0.22	0.34
19	IPI00826289	VCL vinculin isoform 2	49	57	0.03	0.1
20	IPI00718109	TKT Transketolase	91	90	0.11	0.17
21	IPI00691420	TMEM109 Uncharacterized protein		85		0.15
22	IPI01002058	NME1-NME2 protein-like		91		0.15
23	IPI00694142	MIF Macrophage migration inhibitory factor		83		0.32
24	IPI00707040	CTTN Uncharacterized protein		36		0.06
25	IPI00715044	KRT17 Keratin, type I cytoskeletal 17		50		0.16
26	IPI00691584	RPS25 40S ribosomal protein S25		91		0.29
27	IPI00693755	S100A11 Similar to S100 calcium-binding protein A11 (Fragment)		49		0.35
28	IPI00883375	MTAP;PRDX3 PRDX3 protein		63		0.12

29	IPI01004262	LOC100337442 ribosomal protein L35-like		47		0.06
30	IPI00694871	TEKT5 Tektin-5		37		0.07
31	IPI00698932	RPL29 60S ribosomal protein L29		39		0.23
32	IPI00697285	PPIA Peptidyl-prolyl cis-trans isomerase A		37		0.22
33	IPI00694684	SAFB SAFB protein		30		0.04
34	IPI00696746	HDGF Hepatoma-derived growth factor		36		0.14
35	IPI00717276	CARNS1 ATP-grasp domain containing 1		39		0.04
36	IPI00712042	RNH1 Ribonuclease/angiogenin inhibitor 1		36		0.08
37	IPI00694444			33		0.24
38	IPI00693931	H2AFZ Histone H2A.Z		31		0.29
39	IPI00689632	HIST1H2BM Histone H2B type 1-N		115		1.71
40	IPI00867447	- Uncharacterized protein		29		0.07
41	IPI00694938	SERPINH1 Serpin H1	32	33	0.08	0.17
42	IPI00700471	KRT75 Keratin, type II cytoskeletal 75		27		0.06
43	IPI00692681	LRRC59 Leucine-rich repeat-containing protein 59		30		0.23
44	IPI01003460	AHNAK2 AHNAK nucleoprotein 2		20		0.02
45	IPI00905224	C10H14orf38 hypothetical protein LOC617691		26		0.04
46	IPI00706784	RBMX Uncharacterized protein		22		0.09
47	IPI00698589	PGAM1 Phosphoglycerate mutase 1		21		0.13
48	IPI00843025	HNRNPAB Uncharacterized protein		19		0.1
49	IPI00904615	LOC784502 carcinoembryonic antigen-related cell adhesion molecule 1-like		15		0.05
50	IPI00905628	SF3B2 Uncharacterized protein		18		0.04
51	IPI00716394	PRR14L proline rich 14-like		16		0.02

Table S10. List of downregulated proteins in MAC-T Cell supplemented with L-Tryptophan.

	Protein ID	Protein name	Score		%emPAI (Semi quantification)	
			Con	Trp	Con	Trp
1	IPI01002905	HSPD1 60 kDa heat shock protein, mitochondrial	463		1.04	
2	IPI00708046	TUBB2B Tubulin beta-5 chain	645	247	1.39	0.44
3	IPI00706002	ANXA2 Annexin A2	322	257	0.45	0.2
4	IPI00706594	TUBB2C Tubulin beta-2C chain	321		1.06	
5	IPI00717884	ATP5B ATP synthase subunit beta, mitochondrial	336	129	0.29	0.07
6	IPI01028095	HIST1H2BB Histone H2B	176		1.53	
7	IPI00704788	VGF Neurosecretory protein VGF	227	48	0.53	0.24
8	IPI00715690	CTNNA1 Catenin alpha-1	147	158	0.08	0.04
9	IPI00696554	HNRNPK Heterogeneous nuclear ribonucleoprotein K	163	143	0.33	0.15
10	IPI00712775	EEF1A1 Elongation factor 1-alpha 1	152		0.24	
11	IPI00694107	PFN1 Profilin-1	163	107	0.99	0.26
12	IPI00699280	RPSA Similar to 40S ribosomal protein SA (Fragment)	133		0.76	
13	IPI00971572	FLNA filamin-A	311	38	0.11	0.03
14	IPI00692247	HSPA9 Stress-70 protein, mitochondrial	160	114	0.16	0.05
15	IPI01004083	LOC784131 eukaryotic translation elongation factor 1 alpha 1-like		98		0.07
16	IPI00788547	PLS3 Plastin-3	135	61	0.23	0.11
17	IPI00717376	CKAP4 Uncharacterized protein	67	42	0.12	0.06
18	IPI00694295	ATP5A1 ATP synthase subunit alpha, mitochondrial	139	52	0.27	0.06
19	IPI00688960	SFPQ Uncharacterized protein	50		0.05	
20	IPI00695506	LCP1 Lymphocyte cytosolic protein 1	90		0.23	
21	IPI00708921	KRT18 keratin, type I cytoskeletal 18	66		0.25	
22	IPI01003354	LOC100336381 eukaryotic translation elongation factor 1 alpha 1-like	63		0.15	
23	IPI00696935	HNRNPD Uncharacterized protein	48		0.1	
24	IPI00841695	CCT4 T-complex protein 1 subunit delta	123	39	0.13	0.06
25	IPI00691963	CALR Calreticulin	90	47	0.16	0.08
26	IPI00698793	EIF4A1 Eukaryotic initiation factor 4A-I	84		0.26	
27	IPI01001122	SAP130 Sin3A-associated protein, 130kDa isoform 1	53		0.03	
28	IPI00845184	KRT6A KRT6A protein	70		0.06	
29	IPI00707751	EEF2 Elongation factor 2	48	38	0.12	0.04

30	IPI01001319	LOC787289 heterogeneous nuclear ribonucleoprotein A1-like	34		0.11	
31	IPI00717685	STIP1 Stress-induced-phosphoprotein 1	38		0.19	
32	IPI00712133	FASN Fatty acid synthase	77		0.01	
33	IPI00842450	LARP4 Uncharacterized protein	55		0.05	
34	IPI00706632	EEF1G Elongation factor 1-gamma	44		0.07	
35	IPI00706541	SLC25A3 Isoform B of Phosphate carrier protein, mitochondrial	40		0.09	
36	IPI00734138	HNRNPH1 Uncharacterized protein	34		0.07	
37	IPI00699956	KPNA2 Uncharacterized protein	35		0.06	
38	IPI00722271	NUCB1 Nucleobindin-1	25		0.07	
39	IPI00717000	LOC617264 Uncharacterized protein	31		0.12	
40	IPI00685482	PYGL Glycogen phosphorylase, liver form	38		0.04	
41	IPI00693977	RPN2 Dolichyl-diphosphooligosaccharide--protein glycosyltransferase subunit 2	44		0.05	
42	IPI00686546	CCT2 T-complex protein 1 subunit beta	33		0.06	
43	IPI00839060	ATP1A2 Sodium/potassium-transporting ATPase subunit alpha-2	33		0.03	
44	IPI00693645	CLIC1 Chloride intracellular channel protein 1	36	19	0.3	0.14
45	IPI00714446	GPI Glucose-6-phosphate isomerase	48		0.06	
46	IPI00697851	KRT5 Keratin, type II cytoskeletal 5	32		0.06	
47	IPI00707101	AHSG Alpha-2-HS-glycoprotein	31		0.1	
48	IPI01003325	SYNCRIP synaptotagmin binding, cytoplasmic RNA interacting protein isoform 1	29		0.06	
49	IPI00697691	EPB41L2 erythrocyte membrane protein band 4.1-like 2	27		0.03	
50	IPI00693975	ANKRD35 Uncharacterized protein	27		0.03	
51	IPI00686837	SGOL1 Shugoshin-like 1	24		0.06	
52	IPI00714992	PRKG2 cGMP-dependant type II protein kinase	25		0.04	
53	IPI00707814	TCAM1 Testicular cell adhesion molecule 1 homolog	23		0.06	
54	IPI00710895	PGK1 Phosphoglycerate kinase 1	34		0.08	
55	IPI00688640	RPL11 60S ribosomal protein L11	24		0.19	
56	IPI00714747	SSFA2 Uncharacterized protein	15		0.03	
57	IPI00690007	SAE1 SUMO-activating enzyme subunit 1	19		0.1	
58	IPI00685510	APP Amyloid beta (A4) protein	17		0.05	
59	IPI00695732	RPS2 40S ribosomal protein S2	18		0.12	

Table S11. Amino acid profile of lactogenic medium (mM)

Arginine	0.70
Cysteine	0.10
Glutamine	2.50
Glycine	0.25
Histidine	0.15
Isoleucine	0.42
Leucine	0.45
Lysine	0.50
Methionine	0.12
Phenylalanine	0.22
Serine	0.25
Threonine	0.45
Tryptophan	0.04
Tyrosine	0.21
Valine	0.45

Table S12. PCR cycling conditions used to analyze CSN2 expression.

PCR cycling step	Temperature	Time	Cycle
Initial Incubation	95°C	3 min	1
Denaturation	95°C	10 sec	50
Annealing	55°C	15 sec	1
Extension	72°C	30 sec	1
